# Hepatoprotective Effects of *Morinda citrifolia* Leaf Extract on Ovariectomized Rats Fed with Thermoxidized Palm Oil Diet: Evidence at Histological and Ultrastructural Level

**DOI:** 10.1155/2019/9714302

**Published:** 2019-11-07

**Authors:** C. L. G. Chong, F. Hussan, F. Othman

**Affiliations:** ^1^Department of Anatomy, Faculty of Medicine, Pusat Perubatan Universiti Kebangsaan Malaysia, Jalan Yaacob Latif, Bandar Tun Razak, Cheras, 56000 Kuala Lumpur, Malaysia; ^2^Human Biology Division, School of Medicine, International Medical University, 126 Jalan Jalil Perkasa 19, Bukit Jalil 57000 Kuala Lumpur, Malaysia

## Abstract

*Morinda citrifolia* (Rubiaceae) or Noni was previously reported to have leaf with broad therapeutic property whereas the fruit was rarely described as medicinal. Ironically, extensive research and review has been done on the fruit and little was known about the therapeutic activity of the leaf as a medicinal food. The aim of this study was to investigate the therapeutic effects of *Morinda citrifolia* (MC) ethanolic leaf extract on the hepatic structure and function in postmenopausal rats fed with thermoxidized palm oil (TPO) diet. Thirty eight female Sprague Dawley rats were divided into five groups: sham (Sham), ovariectomized (OVX), ovariectomized and treated with simvastatin 10 mg/kg (OVX+ST), ovariectomized and supplemented with low dose MC 500 mg/kg (OVX+MCLD), and ovariectomized and supplemented with high dose MC 1000 mg/kg (OVX+MCHD). All the ovariectomized groups were fed with TPO diet whereas the Sham group was fed with normal diet. Consumption of TPO diet in postmenopausal rats resulted in obesity, significantly elevated (*P* < 0.05) liver oxidative stress marker; malondialdehyde (MDA), diffuse microvesicular steatosis, and defective mitochondria. Treatment with MC leaf extract prevented hepatic steatosis by significantly increasing (*P* < 0.05) the liver antioxidant enzyme SOD and GPx, significantly increasing (*P* < 0.05) ALP, decreasing liver lipids infiltration, preventing mitochondrial damage, and overall maintaining the normal liver histology and ultrastructure. In conclusion, we provided detailed histological and ultrastructural evidence showing hepatoprotective effects of MC leaf extract through its antioxidant mechanism.

## 1. Introduction

Hepatic steatosis is a pathological condition that is prevalent in postmenopausal women due to loss of protective effects of oestrogen.Oestrogen deficiency that occurs following menopause causes metabolic changes, alteration in the body composition, and body fat distribution that leads to liver lipid infiltration [1]. Previous animal studies demonstrated that ovariectomy resulted in progressive fat accumulation in the liver [2]. Consumption of thermally oxidized oil or thermoxidized palm oil (TPO) diet by postmenopausal subjects appeared to accelerate the development of hepatic steatosis [2]. TPO is commonly present in daily food especially in fried cuisine and processed food [3]. The cooking oil is reused repeatedly in order to save costs. Chronic consumption of TPO is hazardous to health especially in elderly postmenopausal women because repeated heating of the oil at high temperature decreases the antioxidant content in the oil, increases lipid peroxidation, and generates free radicals-induced oxidative stress in the liver [4]. Previous animal studies showed that ingestion of food containing TPO resulted in elevated liver enzyme and microsteatosis changes in the liver [5]. Currently, there is no effective pharmacological treatment for this pathological condition except for the management of metabolic risk factors by using statins, weight loss, and exercise, but it is unrealistic as it is difficult to achieve or maintain [6]. The use of hormone replacement therapy (HRT) may be beneficial, but it is not recommended for hepatoprotection as it increases the risk of cardiovascular events [7]. Thus, novel therapeutic strategies and nutritive supplementation with functional food are needed to promote liver health.

Noni leaf is the leaf of *Morinda citrifolia* L. (Rubiaceae) or MC leaf which is an edible famine food and medicinal tropical plant originated from Southeast Asia, Australasia, Pacific Islands, and Hawaii [8]. MC is considered as a sacred plant as it was cited in the ancient text as “*Ashyuka*” which in Sanskrit means “longevity” (Neal, 1965). Previous review has reported that the leaf is the most commonly used part of the plants for treatment whereas the fruit is rarely described as medicinal [9]. The leaf is consumed as a raw vegetable by various culture around the world and is also cooked to promote postpartum health [10]. In Malaysia, MC is popularly known as *mengkudu* and is primarily grown for the use in Traditional Malay Medicine to treat a wide range of diseases such as beri beri, fever, cough, liver and kidney diseases, and internal bleeding [11]. MC leaf is rich in nutritient and was included in the World Health Organization (WHO) and Food and Agriculture Organization (FAO) food composition table for East Asia and the Pacific Islands [12]. It was reported to have a higher level of *β*-carotene compared to other green leafy vegetables and have successfully cured night blindness in children [13]. Rare phytoactive substances with health promoting potential isolated from the leaf includes dehydromethoxygaertheroside, dehydroepoxymethoxygaertheroside, borreiagenin [14], citrifoside, pheophorbide A, pyropheophorbide A, ursolic acid [15], and flavanoids [16]. Previously, we have reported that MC leaf extract possesses antiatherosclerotic effect through its anti-inflammatory activity in the aorta [17]. Since the liver is an indicator of vascular health by secreting and regulating various molecular cardiovascular disease (CVD) risk factors, we further investigated the mechanism of action of MC leaf by looking into the detailed histological and ultrastructural changes in the liver [18]. In this study, we investigated the basis of using MC leaf as a medicinal food in Traditional Malay Medicine to prevent liver disease by studying the effects of the leaf extract supplementation on the liver of postmenopausal rats fed with thermoxidized palm oil (TPO) diet. In particular, we studied the metabolic indicators (body weight, dietary intake, and 11*β*HSD1), liver function (transaminase level and antioxidant enzyme), and oxidative stress marker (MDA) with emphasis on the liver histological and ultrastructural findings. To the best of our knowledge, there is no other study done on the effect of MC on the ultrastructure of the liver.

## 2. Material and Methods

### 2.1. Preparation of *Morinda citrifolia* Ethanolic Leaf Extract

*Morinda citrifolia* ethanolic leaf extract in powder form was prepared by Professor Suhaila Mohamed from the Department of Bioscience, Universiti Putra Malaysia. Voucher specimen is available at the herbarium of the department. The extract was prepared by the following procedure as described by the manufacturer. Fresh *Morinda citrifolia* leaf were collected from Bukit Expo, Universiti Putra Malaysia, and was identified by a botanist. The leaves were washed and homogenized with water. Equal volume of 70% ethanol was then added, soaked for 3 hours, and filtered. The filtrate was put into rotary evaporator to remove the solvent. The resultant green paste was added with 20% starch to make it into powder form and dried in oven. The dried extract was packed in polythene bags with nitrogen purge. The extract was administered via oral gavage daily for three months at the doses of 500 mg/kg and 1000 mg/kg to the respective treatment groups [19].

### 2.2. Preparation of Thermoxidized Palm Oil Diet

Thermoxidized palm oil diet was custom prepared in our laboratory by formulating 5 times heated palm oil (15% *w*/*w*) with standard rat chow [20]. Fresh palm oil (Lam Soon Edible Oil, Malaysia) was thermally oxidized by heating it for five times through frying process [21]. Briefly, 2.5 litres of fresh palm oil was heated in a stainless-steel deep fryer until the temperature reached 180°C after which 1 kg of sweet potatoes were added and fried for 10 minutes. After the frying process, the palm oil was left to cool down to room temperature for 5 hours. The same oil was reused to fry the next batch of sweet potatoes without adding any fresh palm oil. The whole frying process was repeated four times to obtain 5 times heated palm oil (5HPO). 15% weight/weight of the prepared oil was mixed with ground standard rat chow (Gold Coin Sdn Bhd, Malaysia) and then stored in a tight container. The test diet was prepared fresh daily, weighed, and fed to the rats for 3 months.

### 2.3. Experimental Animals

Thirty eight healthy female Sprague Dawley rats (*n* = 38) aged 6 months old with body weight of 250-300 g were obtained from the Laboratory Animal Resource Unit, Universiti Kebangsaan Malaysia. The rats were housed in individual plastic cages at room temperature (27°C ± 2°C) with adequate ventilation and a 12-hour light-dark cycle in the Anatomy Department Animal House. All the experimental animals had *ad libitum* access to food (rat chow from Gold Coin, Selangor Malaysia) and tap water. All the animal handling procedures were in accordance with the institutional animal ethical guidelines with ethical approval number (UKMAEC approval number: FP/ANAT/2014/KHIN/24-SEPT./610-SEPT.-2014-JUNE-2016).

### 2.4. Study Design

The rats were acclimatized for one week and provided with standard rat chow and tap water. The rats were randomly divided into five groups. The first group underwent mock surgery by opening of the abdominal cavity and sewing it back to simulate surgical stress (Sham, *n* = 7) while the other four groups were ovariectomized (surgical removal of ovaries bilaterally) to produce oestrogen-deficient state. The second group was ovariectomized and fed with thermoxidized palm oil diet (OVX, *n* = 7). The third group was ovariectomized, fed with thermoxidized palm oil diet, and supplemented with oral simvastatin suspended in tap water at the dose of 10 mg/kg/day (OVX+ST, *n* = 8) [22]. The fourth group was ovariectomized, fed with thermoxidized palm oil diet, and supplemented with *Morinda citrifolia* low dose 500 mg/kg (OVX+MCLD, *n* = 8). The fifth group was ovariectomized, fed with thermoxidized palm oil diet, and supplemented with *Morinda citrifolia* high dose 1000 mg/kg (OVX+MCHD, *n* = 8) [19].

Bilateral ovariectomy was performed under anaesthesia using ventral approach [23]. Briefly, a lower abdomen midline skin incision was made; the ovary and part of the oviduct was identified, exteriorized, and removed. The same process was repeated to remove the contralateral ovary. The incision on the abdominal musculature was closed with 4/0 absorbable catgut suture (Merck, Germany) followed by closure of the skin incision using 4/0 nonabsorbable silk suture (Merck, Germany). Postoperatively, the rats were given antibiotic enrofloxacin (Baytril, Korea) intramuscularly, placed in a clean cage without wood shaving to avoid wound contamination, and strictly monitored postoperatively for behavioural changes. After three weeks of postoperative recovery period, all the ovariectomized rats were fed with thermoxidized palm oil diet and treated for three months. Physiological parameters such as body weight, food intake, and water intake were done to monitor the metabolic changes of the rats. At the end of the experimental period, the rats were sacrificed with diethyl ether (Sigma-Aldrich, Germany). The blood and liver tissues were collected. The success of ovariectomy was confirmed at necropsy by observation of marked atrophy of the uterine horns.

### 2.5. Serum Biochemical Analyses

Whole blood samples were collected via cardiac puncture, placed into plain tube, and sent immediately to Pathlab & Clinical Laboratory Sdn. Bhd., Malaysia, for serum analyses of liver function test (LFT). Serum AST, ALT, and ALP were measured using assay kits by colorimetric method according to the manufacturer's guidelines.

### 2.6. Liver Tissue Oxidative Stress Assessment

Immediately after sacrificing the rats, the liver tissues were dissected and stored at −80°C for detection of antioxidant enzymes. A part of the liver tissues were also excised and fixed for histological staining.

MDA level was measured using lipid peroxidation (MDA) colorimetric/fluorometric assay kit by BioVision, USA. 11*β*-Hydroxysteroid dehydrogenase enzyme type 1 (11*β*HSD1) was measured using ELISA kit for 11*β*HSD1 (Cloud-Clone Corp, USA). Tissue glutathione (GSH) was measured by using glutathione assay kit by Cayman Chemical Company, USA [24]; glutathione peroxidase (GPx) was measured using glutathione assay kit by Cayman Chemical Company, USA (Forstrom & Wheeler, 1990); catalase (CAT) was measured using catalase assay kit by Cayman Chemical Company, USA [25]; and superoxide dismutase (SOD) was measured using superoxide dismutase askay Kit by Cayman Chemical Company, USA [26]. All procedures were done according to the manufacturers' guidelines.

### 2.7. Histological Analyses and Histomorphometry

Immediately after removal, the liver tissues were fixed in 10% formalin for a week with a change in formalin solution to remove traces of blood from the tissue for histological staining. The samples were dehydrated and embedded in paraffin. Thin sections (5 *μ*m) of the liver was cut and stained with haematoxylin and eosin stain to detect the presence of steatosis [27]. The tissues were also stained with Verhoeff van Gieson (VVG) stain to detect the presence of thinning and disruption of the elastic fibres [28].

In qualitative electron microscopy study, 1 mm^3^ sections of the liver tissues were obtained from two rats from each group. They were rinsed with 0.1 M phosphate-buffered saline (PBS), fixed with glutaraldehyde fixative, and stored at 4°C. The tissues were rinsed again with 0.1 M PBS followed by secondary fixation using 3% uranyl acetate and dehydration with series of ethanol. Infiltration process was done in propylene oxide and embedded in resin at 60°C for 24 hours. After the resin polymerized, the samples were sectioned with a glass knife and stained with toluidine blue stain for semithin section. The area of interests in the semithin tissue samples were identified. Ultrathin sections of the area of interests were obtained using a diamond knife. The samples were placed on the copper grid size of 200 networks. The results were viewed by two expert observers in a double-blinded fashion under transmission electron microscope Tecnai G2 model [29].

### 2.8. Statistical Analysis

All data were presented as mean ± standard error (SEM). Statistical significance level was set as *P* < 0.05. Normally distributed data were analysed by parametric test using analysis of variance (ANOVA) followed by post hoc Tukey. All statistical analyses were performed by using Statistical Package for Social Sciences (SPSS) software version 22 (SPSS Inc., Chicago, IL, USA).

## 3. Results

### 3.1. Metabolic Function

Obese postmenopausal rat models were established two weeks after the ovariectomy. All the ovariectomized rats have body weight greater than the mean body weight plus one fold of standard deviation of the normal Sham operated group. Body weight of OVX (292 ± 5 g), OVX+ST (291 ± 8 g), OVX+MCLD (305 ± 11 g), and OVX+MCHD (294 ± 12 g) were significantly higher (*P* < 0.05) than the Sham group (249 ± 5 g). However, there were no significant differences (*P* > 0.05) in the body weight among all the ovariectomized groups. Food intake of OVX (16 ± 0.65 g/day), OVX+ST (16 ± 0.38 g/day), OVX+MCLD (15 ± 0.48 g/day), and OVX+MCHD (15 ± 0.65 g/day) were significantly higher (*P* < 0.05) than that of the Sham group (13 ± 0.34 g/day). OVX+ST (21.5 ± 0.68 ml/day) was shown to have significantly lower water intake compared to the Sham group (25.57 ± 0.90 ml/day). 11*β*-Hydroxysteroid dehydrogenase enzyme type 1 (11*β*HSD1) revealed no significant difference (*P* > 0.05) in all groups. The data is summarized in Table 1.

### 3.2. Serum Biochemical Parameters (Liver Function)

No significant difference (*P* > 0.05) were noted in the liver weight in all groups. Serum markers of liver function showed no significant difference (*P* > 0.05) in the liver transaminase (AST and ALT) in all groups. Isolated rise of ALP (*P* < 0.05) were seen in OVX+ST (18.78 ± 1.69 U/mL) and OVX+MCHD (18.27 ± 2.03 U/mL). The data is summarized in Table 1.

### 3.3. Oxidative Stress Assessment

Consumption of thermoxidized palm oil diet were shown to significantly elevate (*P* < 0.05) the level of lipid peroxidation product malondialdehyde (MDA) in the untreated OVX group (7.54 ± 0.62 nmol/mg) and OVX+MCLD (8.31 ± 0.32 nmol/mg) as compared to the Sham group (5.74 ± 0.48 nmol/mg). The group supplemented with high dose MC (OVX+MCHD) showed significantly increased (*P* < 0.05) GPx level (44.53 ± 2.50 nmol/mg) compared to Sham (27.92 ± 1.78 nmol/mg), OVX (27.28 ± 3.51 nmol/mg), and OVX+MCLD (32.35 ± 1.36 nmol/mg). In addition, the OVX+MCHD group also showed significantly higher (*P* < 0.05) level of SOD (0.10 ± 0.01 U/mg) compared to the untreated OVX (0.05 ± 0.01 U/mg). However, no significant differences (*P* > 0.05) were observed in the level of GSH and CAT in all groups. The data is summarized in Table 1.

### 3.4. Liver Histopathological and Ultrastructural Assessment

**Figure 1:**
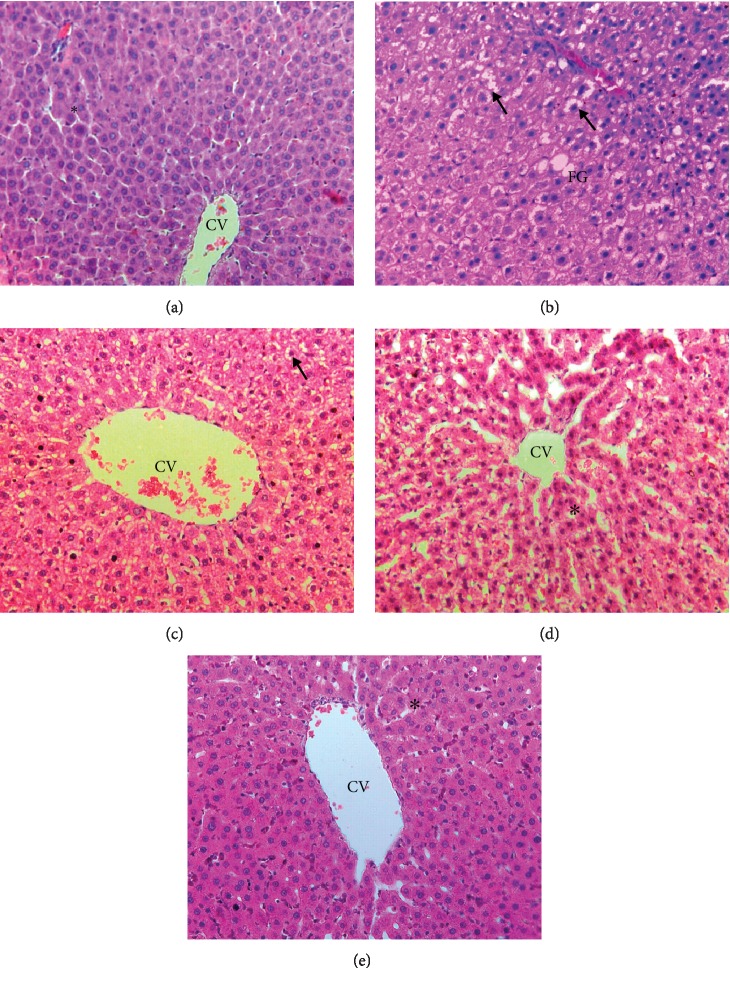
(a) Photomicrograph showing H&E-stained liver tissue of the Sham group. Normal sheets of hepatocytes (^∗^) were seen surrounding the central vein (CV). H&E staining 200x. (b) Photomicrograph showing H&E-stained liver tissue of the untreated OVX group. Note the presence of fat globules (FG) and enlarged hepatocytes with hypercellularity (arrow). H&E staining 200x. (c) Photomicrograph showing H&E-stained liver tissue of the ovariectomized group fed with TPO diet and treated with statin (OVX+ST) which also showed the presence of enlarged hepatocytes (arrow). H&E staining 200x. (d) Photomicrograph showing H&E-stained liver tissue of the ovariectomized group fed with TPO diet and treated with MC leaf 500 mg/kg (OVX+MCLD). Normal sheets of hepatocytes (^∗^) were seen surrounding the central vein (CV). H&E staining 200x. (e) Photomicrograph showing H&E-stained liver tissue of the ovariectomized group fed with TPO diet and treated with MC leaf 1000 mg/kg (OVX+MCHD). Normal sheets of hepatocytes (^∗^) were seen surrounding the central vein (CV). H&E staining 200x.

Histopathological evaluation of the liver showed normal hepatic architectures present in the Sham, OVX+MCLD, and OVX+MCHD where there were no signs of inflammation around the central vein and the surrounding sheets of hepatocytes (Figures 1(a), 1(d), and 1(e)). The untreated OVX group showed pathological features of diffuse microvesicular steatosis with features of hypercellularity, congestion, distortion of sinusoids, enlarged hepatocytes, and fat globules deposition (Figure 1(b)). OVX+ST also showed the presence of enlarged hepatocytes (Figure 1(c)).

**Figure 2:**
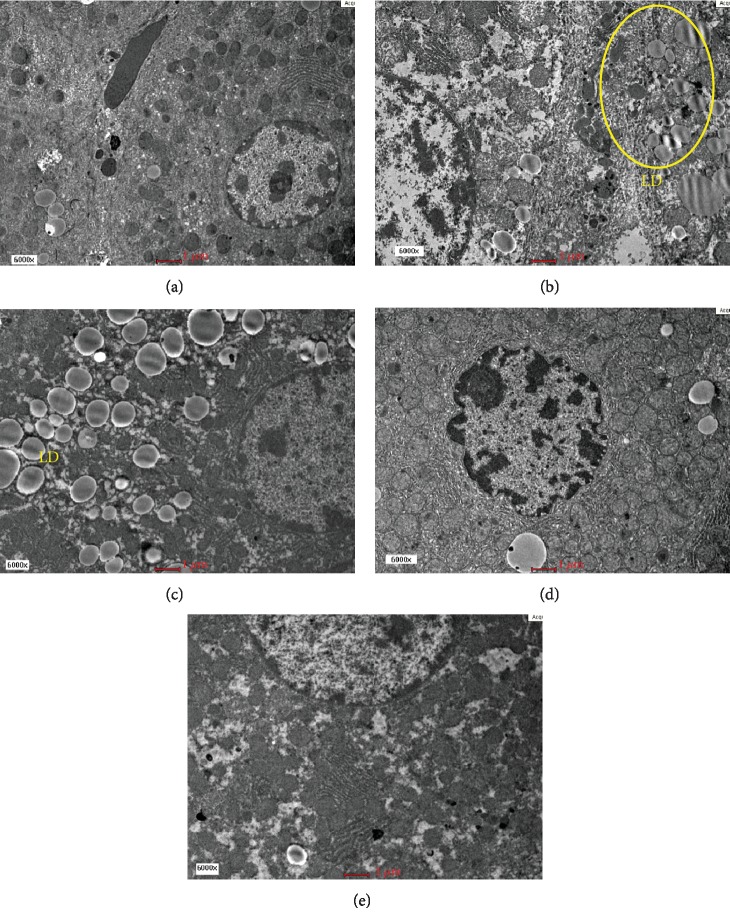
(a) Electron micrograph showing the hepatocyte of the Sham group. Normal organelles were seen. EM 6000x. (b) Electron micrograph showing the hepatocyte of the untreated OVX group. Massive amounts of lipid droplets (LD) accumulation (circle) were present around the relatively enlarged nucleus. EM 6000x. (c) Electron micrograph showing the hepatocyte of the OVX rats fed with TPO diet and treated with statin (OVX+ST). Lipid droplet (LD) accumulation was seen surrounding the nucleus. EM 6000x. (d) Electron micrograph showing the hepatocyte of the OVX rats fed with TPO diet and treated with MC leaf 500 mg/kg. Relatively less lipid droplets were observed. EM 6000s. (e) Electron micrograph showing the hepatocyte of the OVX rats fed with TPO diet and treated with MC leaf 1000 mg/kg (OVX+MCHD). Limited amounts of lipid droplets were present. EM 6000x.

**Figure 3:**
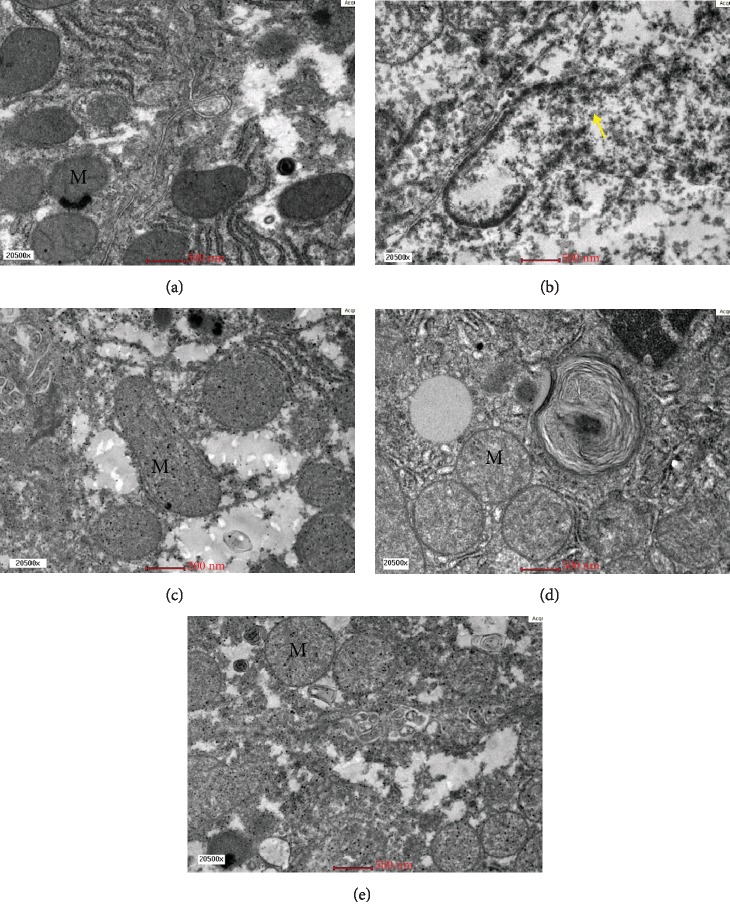
(a) Electron micrograph showing the presence of normal mitochondria with cristae (M) in the hepatocyte of the Sham group. EM 20500x. (b) Electron micrograph showing megamitochondria with cristolysis and mitochondrial rupture (arrow) in the untreated OVX group. EM 20500x. (c) Electron micrograph showing megamitochondria with cristolysis and mitochondrial rupture (arrow) in the untreated OVX group. EM 20500x. (d) Electron micrograph showing normal mitochondria with cristae (M) in the hepatocyte of OVX+MCLD comparable to the Sham group. EM 20500x. (e) Electron micrograph showing normal mitochondria with cristae (M) in the hepatocyte of OVX+MCHD comparable to that of the normal Sham group. EM 20500x.

Qualitative electron microscopic findings revealed normal hepatocytes ultrastructure seen in the Sham group with the presence of normal organelles without necrotic cell, disintegrating cell, and apoptotic body. A few lipid droplets were present in a relatively normal distribution. The untreated OVX group showed pathological features of microvesicular steatosis evidenced by the presence of massive amounts of electron-dense lipid droplets deposition. The nucleus appeared relatively enlarged compared to the Sham group and foamy cytoplasm with dense granular deposits were observed (Figure 2(b)). OVX+ST also showed features of microvesicular steatosis as massive numbers of lipid droplets deposition were noted (Figure 2(c)).The OVX+MCLD and OVX+MCHD groups showed obviously less lipid droplets infiltration comparable to that of the Sham group (Figures 2(d) and 2(e)). Ultrastructural studies revealed normal mitochondria with cristae were present in the Sham group (Figure 3(a)). The untreated OVX group showed megamitochondria and ruptured mitochondria with cristolysis (Figure 3(b)). The OVX+ST also showed elongated mitochondria or megamitochondria (Figure 3(c)), whereas the groups treated with MC showed absence of mitochondrial damage (Figures 3(d) and 3(e)).

## 4. Discussion

Ovariectomized rats fed with thermoxidized palm oil (TPO) diet were used in this study as an experimental model of hepatic steatosis. Ovariectomized rat is an excellent animal model that represent postmenopausal oestrogen deficiency in human. The rats were fed with TPO diet to reflect the actual diet in human where most of our foods are cooked using palm oil especially fried cuisine and processed food [3]. In reality, elderly postmenopausal subjects exposed to TPO diet are more susceptible to develop hepatic steatosis due to loss of protective effects of oestrogen [2].

After 12 weeks of TPO feeding, all the ovariectomized rats developed hyperphagia and obesity. This metabolic change is due to the removal of catabolic actions of oestrogen which act upon central neuropeptidergic pathway that regulate feeding and energy expenditure in the hypothalamus [30]. In obese subjects, failure to downregulate 11*β*-HSD1 enzyme causes liver lipids infiltration [31]. However, in this study, we did not observe any significant difference in the level of 11*β*-HSD1 enzyme in all groups. According to this findings, we concluded that 11*β*-HSD1 did not play a role in the pathogenesis of hepatic steatosis in rat models.

Consumption of TPO in postmenopausal rats did not cause significant increase in liver transaminase which indicate that the liver is functioning optimally and there is no acute liver toxicity present. This findings were in contrast with previous study by [5]. The discrepancy occurs because longer duration of TPO feeding was used in that study. Isolated rise in ALP which were noted in the groups treated with statin (OVX+ST) and OVX+MCHD that could indicate increase in bone formation. Both statin and MC were reported to have significant antiosteoporotic activity by increasing the expression of ALP in vitro and increasing osteoclasts activity [32, 33].

Consumption of TPO in postmenopausal rats leads to oxidative stress in the liver. The untreated OVX group showed significantly higher lipid peroxidation product MDA. This result is in accordance with [34]. Repeated heating of palm oil at high temperature decreases the antioxidant content of the oil and changes its chemical composition through hydrolysis, oxidation, and polymerization [4]. Hydrolysis of the oil molecule produces free fatty acid (FFA) and secondary lipid peroxidation products such as aldehydes, ketones, and alcohols. Oxidation of lipids generates free radicals as fatty acid undergoes saturation and receives reactive oxygen species (ROS). ROS from the oil is absorbed into the food and subsequently into the GIT and blood circulation where it damages the lipids by initiating lipid peroxidation. The end product of lipid peroxidation is MDA which is highly mutagenic. In the liver, MDA causes inflammation [35] and oxidative stress leading to hepatic steatosis [36]. In our study, the group treated with low dose MC also showed significantly higher MDA level probably because the low dose was insufficient to promote therapeutic effects. However, high-dose MC showed lower MDA level nonsignificantly compared to the untreated group. Treatment with high-dose MC showed significantly higher antioxidant enzyme GPx and SOD in accordance with those reported by [37]. These findings proved that MC leaf extract protects the liver from oxidative stress by increasing the antioxidant enzyme, thus maintaining the oxidative balance in the liver. However, treatment with high-dose MC did not cause significant increase in CAT and GSH.

Oxidative stress is manifested as microvesicular steatosis visualized under H&E staining in the untreated OVX group. Microvesicular steatosis indicates the presence of severe mitochondrial dysfunction [38] due to a defect in mitochondrial *β*-oxidation [39]. In this study, TPO acts as a hepatotoxin that initiates lipid peroxidation causing histological changes such as distended hepatocytes, clear cytoplasm instead of pink, centrally located nucleus, and hepatocytes ballooning or enlarged hepatocytes. Hepatocytes ballooning is a histological hallmark of cellular injury and cytoskeletal damage [38]. Treatment with MC leaf minimalized all these histological damages.

Under electron microscopy, the most striking features found in the untreated OVX group include massive amounts of lipid droplets accumulation, foamy cytoplasm, matrix granulation, and ruptured mitochondria (Figures 2(b) and 2(c)). Megamitochondria and ruptured mitochondria indicate the presence of biochemical hepatic injury due to the disturbance in the mitochondrial electron transport chain and oxidative injury [40]. Megamitochondria also represents cellular adaptive response to oxidative damage. Elongated and enlarged mitochondria indicate the presence of metabolic abnormality [38]. Decreased protein synthesis in the mitochondria and impaired respiratory chain function lead to the appearance of mitochondrial matrix granules. Foamy cytoplasm was prominently seen in the untreated OVX group due to glycogen accumulation which occurs when lipids accumulate in the hepatocytes causing hepatocyte swelling, narrowing of sinusoidal lumen, sinusoidal damage, and decreased blood flow [29].

Treatment with statin in the absence of dyslipidemia appeared to cause massive accumulation of lipid droplets in the liver (Figure 2(c)) and megamitochondria (Figure 3(c)). Based on these findings, we do not support the use of statin as a primary prevention or prophylaxis of cardiovascular disease (CVD) as it causes liver lipid infiltration [41]. Treatment with MC did not cause lipid accumulation in the liver and mitochondrial damage (Figures 2(d) and 2(e)). These ultrastructural findings justified that MC leaf extract possesses hepatoprotective effects by preventing liver lipid accumulation, minimalized hepatocellular damage, and overall maintaining the normal histology of the liver.

Our findings are in contrast with previous reports stating that anthraquinones found in MC: morindin and rubiadin, are toxic and all MC products are screened for the presence of these compounds [8]. However, the toxic anthraquinones are only found in the root and bark where it is used as a colouring dye and not in the leaf [11]. Recent findings demonstrated that MC leaf extract showed no observable hepatotoxicity [42]. Phytoactive substances responsible for the antioxidant effects observed in this study are flavanoids (rutin, quercetin, and kaempferol) which act against lipid peroxidation, nitric oxide, and hydroxyl radicals [43]. Flavanoids found in MC also exert anti-inflammatory activity by inhibiting the release of proinflammatory cytokines such as TNF-*α*, IL-1*β*, and NO [44]. Ursolic acid also played a vital role in reversing hepatic steatosis and improving metabolic function by upregulating the hepatic peroxisome proliferator-activated receptor (PPAR-*α*) [45].

## 5. Conclusion

To date, our study is the first to our knowledge to rationalize the hepatoprotective effects of MC leaf extract against hepatic steatosis at ultrastructural level. Consumption of TPO diet in postmenopausal rats resulted in adverse metabolic changes such as obesity and hyperphagia, elevated lipid peroxidation product, MDA in the liver, and prominent pathological changes in the liver ultrastructure such as diffuse microvesicular steatosis with severe lipid droplet deposition and mitochondrial damage. Treatment with MC leaf extract resulted in elevated liver antioxidant enzymes, less lipid droplet deposition, and the normal liver histology and the ultrastructure was maintained. In conclusion, MC leaf extract prevents cellular hepatic injury through the antioxidant mechanism of flavanoids and ursolic acid.

## Figures and Tables

**Table 1:** Physiological parameters and serum and liver tissue analysis of MCLE treatment.

Variable	Sham	OVX	OVX+ST	OVX+MCLD	OVX+MCHD
Metabolic function					
Body weight (g)	249 ± 5	292 ± 7^∗^	291 ± 8^∗^	305 ± 11^∗^	294 ± 12^∗^
Food intake (g)	12.86 ± 0.34	16.43 ± 0.65^∗^	15.5 ± 0.38^∗^	15.13 ± 0.48^∗^	15.00 ± 0.65^∗^
Water intake (ml)	25.57 ± 0.9	24.29 ± 1.23	21.5 ± 0.68^∗^	21.75 ± 1.22	22.5 ± 0.53
11-*β*HSD1 (ng/ml)	31.9 ± 3.43	36.59 ± 0.42	37.06 ± 0.12	36.13 ± 0.92	33.82 ± 2.68
Liver function					
Liver weight (g)	7.43 ± 0.28	7.64 ± 0.37	6.69 ± 0.41	7.31 ± 0.34	6.69 ± 0.16
AST (U/mL)	128.6 ± 5.31	140.71 ± 17.626	156.13 ± 15.36	182.17 ± 20.56	172.13 ± 15.44
ALT (U/mL)	61.43 ± 6.36	0.71 ± 4.8217.3	54.13 ± 5.54	54.57 ± 3.08	63.43 ± 6.23
ALP (U/mL)	12.04 ± 1.06	1 ± 0.56	18.78 ± 1.69^∗^	13.76 ± 1.44	18.27 ± 2.03^∗^
Oxidative indices					
MDA (nmol/mg)	5.74 ± 0.48	7.54 ± 0.62^∗^	7.30 ± 0.33	8.31 ± 3.32^∗^	6.85 ± 0.31
GSH (*μ*m)	26.27 ± 2.56	30.03 ± 1.52	30.46 ± 1.18	28.74 ± 1.68	34.31 ± 1.71
GPx (nmol/mg)	27.92 ± 1.78	27.28 ± 3.51	35.28 ± 28	32.35 ± 1.36	44.53 ± 2.50^∗^^#+^
SOD (U/mg)	0.064 ± 0.12	0.060 ± 0.08^#^	0.078 ± 0.06	0.067 ± 0.12	0.103 ± 0.014
CAT (nmol/mg)	6.74 ± 0.53	7.16 ± 0.48	7.79 ± 0.59	7.27 ± 0.63	7.29 ± 0.64

Values are mean ± SEM, *n* = 7 (Sham, OVX), *n* = 8 (OVX+ST, OVX+MCLD, OVX+MCHD). ^∗^Significant difference from Sham, ^#^significant difference from OVX, ^+^significant difference from OVX+MCLD (*P* < 0.05).

## Data Availability

All the analysed data were presented in the thesis of Dr. Gloria Chong Chui Lin in the fulfilment of Master in Medical Science and are available at Universiti Kebangsaan Malaysia library. Correspondence should be addressed to miss_gloe@yahoo.com.
